# Longevity Promoted

**Published:** 1859-06

**Authors:** 


### LONGEVITY PROMOTED.

To a very great extent, our life is in our own hands, although it is the prevailing fashion of the times, to regard death, especially if it is premature, or if the person dying of any age, occupies a position of influence and usefulness, as a "mysterious dispensation of Providence," when in reality, "Providence" had nothing to do with it; had no direct agency in the matter; only indirectly, in having founded the laws of our being. When men die short of eighty or an hundred years, it is the result of violated law, and almost always on their own part.

If a sedentary man eats a hearty meal late in the day, or a laborious man does the same thing after long fasting and protracted exertion, ending in great bodily fatigue, and is attacked in the night with cramps, cholic, or cholera morbus, or other form of looseness of bowels, ending in death next morning, there is no "mystery" in that. The man is his own destroyer, and in that destruction, his Maker had no agency.

A man in the prime of life enters a crowded omnibus, after a long or rapid walk, which has induced free perspiration, the air appears alone to him almost suffocating, and with an insanity, resulting from detached scraps of knowledge about the advantages of pure air, he opens the window, and the breeze is delicious; but before he is aware of it he finds himself chilled, and wakes up in the morning with acute throat disease, or inflammation of the lungs or violent fever; or the magazine of impending consumption has been fired, and he wilts, and wastes and dies—by his own hand, from ignorance of the fact, that no air of any coach, or conveyance, or crowded room, is a thousandth part as injurious or dangerous to a new comer, as the purest air that was ever breathed, if it comes with a draft upon one who is perspiring and remains in a still position.

The most talented and useful clergyman in the land, whose influence is widening and deepening every day for good, carrying all before him by the power of his eloquence, but after an unusual effort in which the heart, as well as brain and body, all have been brought into an exhausting requisition, all heat ed, and perspiring and debilitated, he feels it his duty to attend some urgent call, and hastes away into the cold raw damp air, the bleak wind whistling fiercely by, and in a week, in the midst of his usefulness, he is laid in the grave, by peritoneal (abdominal) inflammation, or quinsy, or pleurisy—his own destroyer, for he acted as if he were made of iron, instead of flesh and blood. He threw his life away, in an indistinct impression, that as he was doing a good work, a miracle would be wrought for his protection; and because the laws of nature were allowed to take their usual course, it is deemed a "wonderful and mysterious dispensation of Providence," and we cry "His ways are past finding out."

A woman holds on her lap a lovely child. It was born perfect, fair and beautiful, but the aristocratic mother has not the stamina to feed it, for the natural fountain is short of a full supply, and ale and beer, and the universal milk punch are swilled by the pint and quart a day, to "make milk." But just in proportion as it is alcoholic, it is innutritive, it creates an appearance of flesh, and strength, and thrift, but all as unreal and transient as Jonas' gourd, and the child, by the excitement thrown to the head, dies of water on the brain; or if by virtue of the father's more robust and vigorous constitution and temperament, infancy and youth are survived, the instinct for excitement planted in the first year, wakes up again at maturity, and the young lady wastes her intellect in the stimulus of novel reading, or the young man destroys intellect and body too, in yielding to the fires of liquor and of license; and suddenly as the bank deposit of a spendthrift heir gives out, so suddenly is exhausted the vital force and he dies at his toilet, in his chair, at the table or on the street, of Heart Disease, the coroner's jury reports; a "mysterious dispensation of Providence" is the response from another direction. The true verdict is, "died by a mother's folly, committed twenty years agone!"

Great men are gentle. God is love. His way of removing his children from their lower home, is in tenderness, for he has appointed that in the habitual exercise of moderation, all the parts of the human machine shall wear out equally, one not faster than another; one no sooner than another; all gradually cease; all fail at the same instant; one worn out function does not cease its operation, while another, in its full vigor, strives to go on without it; hence the universally observed fact is, that the very old die gently, without a struggle, and scarce a pang; die as an infant falls to sleep amid its mother's lullaby; "like as a shock of corn cometh in his season."

"So fades a summer cloud away,
So sinks the gale when storms are o'er;
So gently shuts the eye of day,
So dies a wave along the shore."

